# Difference in the regulation of IL-8 expression induced by uropathogenic *E. coli *between two kinds of urinary tract epithelial cells

**DOI:** 10.1186/1423-0127-16-91

**Published:** 2009-10-03

**Authors:** Kun-Wei Tsai, Hong-Thih Lai, Tzung-Chieh Tsai, Yi-Chien Wu, Ya-Ting Yang, Kwei-Yi Chen, Chun-Ming Chen, Yi-Shuan J Li, Cheng-Nan Chen

**Affiliations:** 1 Department of Internal Medicine, Buddhist Dalin Tzu Chi General Hospital, Dalin, Chiayi, Taiwan, Republic of China; 2 Department of Aquatic Biosciences, National Chiayi University, Chiayi 600, Taiwan, Republic of China; 3 Department of Microbiology and Immunology, National Chiayi University, Chiayi 600, Taiwan, Republic of China; 4 Department of Biochemical Science and Technology, National Chiayi University, Chiayi 600, Taiwan, Republic of China; 5 Department of Bioengineering and Whitaker Institute of Biomedical Engineering, University of California, San Diego, La Jolla, CA 92093-0427, USA

## Abstract

Bacterial adherence to epithelial cells is a key virulence trait of pathogenic bacteria. The type 1 fimbriae and the P-fimbriae of uropathogenic *Escherichia coli *(UPEC) have both been described to be important for the establishment of urinary tract infections (UTI). To explore the interactions between the host and bacterium responsible for the different environments of UPEC invasion, we examined the effect of pH and osmolarity on UPEC strain J96 fimbrial expression, and subsequent J96-induced interleukin-8 (IL-8) expression in different uroepithelial cells. The J96 strain grown in high pH with low osmolarity condition was favorable for the expression of type 1 fimbriae; whereas J96 grown in low pH with high osmolarity condition was beneficial for P fimbriae expression. Type 1 fimbriated J96 specifically invaded bladder 5637 epithelial cells and induced IL-8 expression. On the contrary, P fimbriated J96 invaded renal 786-O epithelial cells and induced IL-8 expression effectively. Type 1 fimbriated J96-induced IL-8 induction involved the p38, as well as ERK, JNK pathways, which leads to AP-1-mediated gene expression. P fimbriated J96-induced augmentation of IL-8 expression mainly involved p38-mediated AP-1 and NF-κB transcriptional activation. These results indicate that different expression of fimbriae in J96 trigger differential IL-8 gene regulation pathways in different uroepithelial cells.

## Background

Urinary tract infection (UTI) is one of the most common bacterial infections that affect humans throughout their life span. UTI occurs in every age group, from newborns to the elderly patients; it has the greatest impact on females of all ages (especially during pregnancy), and males as the kidney transplant recipients or with structural abnormalities of the urinary tract. The most common bacterium that causes UTI is uropathogenic *Escherichia coli *(UPEC). These bacteria are sensitive to a variety of environmental cues such as differences in temperature, nutrients, pH, and osmolality [1-3]. Human urine has extreme fluctuations in osmolarity and pH [4,5]. The osmolalities in human urine can range from 0.038 to 1.4 mol/kg, with the osmolarity of the urine in kidneys is much higher than that in bladder [6]. In addition to osmotic variations, the pH of human urine can vary between 5.0 and 8.0, depending on physiological constraints and the diet of the individuals [4-6]. Kidney urine typically has a lower pH than bladder urine because of the dilution effect in the bladder [6].

Adherence and invasion to uroepithelial cells is a critical step in the ability of bacteria to cause UTI. Attachment is regulated through specific interactions between bacterial surface components (adhesins) and host cell receptors. The adhesins of UPEC exist as filamentous surface organelles, termed pili or fimbriae. Fimbrial adhesins are important virulence factors that allow binding of the bacteria to specific receptors on uroepithelial cells [7]. The two adhesins most commonly associated with UTI are type 1 and P fimbriae [8]. Type 1 fimbriae are essential for UPEC colonization of the lower urinary tract [9], whereas P fimbriae are critical for that of the upper urinary tract [10]. To limit immune exposure and inflammation, the expression of type 1 and P fimbriae is phase variable, which the bacteria can switch between different fimbriated states. Type 1 fimbriae are encoded by a *fim *gene cluster, including the adhesin subunit, FimH. The expression of type 1 fimbriae depends on the orientation of the invertible element located between two inverted repeat [11]. This element contains a promoter which increases the expression of the *fim *subunit genes in phase-on orientation. The binding specificity of P fimbriae is determined by the PapG adhesin. Previous work has demonstrated that activated P-fimbrial gene cluster can act on the *fim *locus to prevent expression of type 1 fimbriae by switching the *fim *gene cluster to phase-off orientation [11]. It was previously observed that *E. coli *expresses mainly one fimbrial type at a time [12]. This may be important to limit immune exposure and to prevent the physical interference of one adhesin with another.

Uroepithelial cells function as a physical protective barrier against invasion by UPEC. In addition, they also play a role in local innate immune responses by secreting bioactive substances, such as chemokines, when exposed to pathogens [8]. Interleukin-8 (IL-8), a member of the CXC chemokine family, plays a pivotal role in regulating neutrophil chemotaxis toward sites of infection, and in inducing urinary tract inflammation [13]. Transcriptional regulation of IL-8 is controlled by a tight regulatory signal network, involving the complex interplay of different mitogen-activating protein kinase (MAPK) cascades in several cell types [14,15].

As mentioned above, the environments in kidney and bladder are different, the epithelial cells isolated from kidney and bladder are expected to have differential responses to different adhesins. We hypothesize that signaling pathways lead to IL-8 secretion in kidney and bladder epithelial cells are different. The goal of this study is to elucidate the signaling network that orchestrates expression of IL-8 by UPEC invasion in different cell types. The results demonstrated that UPEC strain J96 grown in different pH and osmolality conditions expresses different fimbriae, and therefore preferentially targets either kidney or bladder uroepithelial cells for IL-8 production. Furthermore, the signaling pathways leads to IL-8 secretion are different in kidney and bladder uroepithelial cells.

## Materials and methods

### Materials

All culture materials were purchased from Gibco (Grand Island, NY, USA). GenomicPrep Cells DNA Isolation Kits were purchased from Amersham Pharmacia Biotech, Inc (Piscataway, NJ). PD98059 (ERK inhibitor), SP600125 (JNK inhibitor), and SB203580 (p38 inhibitor) were purchased from Calbiochem (La Jolla, CA). Mouse monoclonal antibodies (mAB) against extracellular signal-regulated kinase 2 (ERK2), JNK1, phospho-ERK, and phospho-JNK were purchased from Santa Cruz Biotechnology (Santa Cruz, CA). Rabbit polyclonal antibodies against p38 and mouse monoclonal phospho-p38 antibody were purchased from Cell Signaling Technology (Beverly, MA). IL-8 ELISA kit was obtained from R & D Systems (Minneapolis, MN). p38 siRNA and control siRNA (scrambled negative control containing random DNA sequences) were purchased from Invitrogen (Carlsbad, CA). Tanshinone IIA (TIIA) were purchased from Biomol (Plymouth Meeting, PA). Pyrrolidine dithiocarbamate (PDTC) and other chemicals of reagent grade were obtained from Sigma (St Louis, MO).

### Plasmid, bacterial strains and growth conditions

Non-fimbriated *E. coli *strain HB101 and uropathogenic strain J96 (expresses type 1 or P fimbriae) [16] were obtained from American Type Culture Collection (Rockville, MD). Non-fimbriated *E. coli *strain 83972 [17] was a generous gift from Dr. Barbara W. Trautner (Michael E. DeBakey Veterans Affairs Medical Center, Houston, Texas). Plasmid pUC18 expressing PapG II adhesin [18] was a generous gift from Dr. Jiunn-Jong Wu (National Cheng Kung University, Tainan, Taiwan). This plasmid was used to generate P fimbriated transformed *E. coli *83972 in this study.

Bacteria stocks were stored at -20°C in 50% glycerol and broth. All bacteria were cultured in Luria-Bertani (LB) broth overnight at 37°C. Broth consisted of 32 g of tryptone, 20 g of yeast extract, 5 g of NaCl, and 5 mL of 1 N NaOH per liter. Bacterial concentrations were determined by OD 600 nm with each 0.1 OD equal to 10^8 ^bacteria/mL.

To obtain variations in pH in vitro, the pH of LB medium was adjusted by using 0.1 M Na_2_HPO_4_-NaH_2_PO_4 _buffer combined with 1% (vol/vol) glycerol. We prepared LB medium with pHs 5.5 and 7.0 confirmed with a pH meter. The osmolality of pH 5.5 LB broth was adjusted by adding NaCl to final concentration of 400 mM [3]. The pHs of cultures were further checked after overnight incubation.

### Cell culture

The human bladder epithelial cell line 5637 and human renal carcinoma cell line 786-O were obtained from American Type Culture Collection (Rockville, MD). Cells were maintained in RPMI-1640 medium supplemented with 10% FBS.

### Hemagglutination assay and Gal-Gal coated latex bead agglutination

For hemagglutination assays, a 3% (vol/vol) solution of erythrocytes with or without 50 mM mannose was used to determine type 1 fimbrial mannose-sensitive hemagglutination. Approximately 1 × 10^9 ^CFU of J96 bacteria from broth were serially diluted twofold in 96-well microtiter plates. An equal volume of erythrocyte solution was mixed with the bacterial suspension. A diffuse mat of cells across the bottom of the well indicated positive hemagglutination [19]. For Gal-Gal latex bead agglutination, latex beads coated with α-Gal(1-4)β-Gal were used to determine the presence of P fimbria by latex agglutination. Approximately 1 × 10^9 ^CFU of bacteria cultured in broth in a total volume of 10 μL, was mixed with 25 μL PBS and 2 μL latex beads in a 96-well microtiter plate. A granular settling of latex beads on the bottom of the well indicated positive latex agglutination [19].

### Invasion assays

The epithelial cell lines 5637 and 786-O were seeded into 24-well plates and grown to confluence. Just before infection, the cell culture medium was replaced with fresh medium. Cells were infected with a multiplicity of infection (MOI) of 20 bacteria per host cell. After 1 h incubation at 37°C, cells were washed twice with PBS and then incubated for another 2 h in medium containing 100 μg/mL membrane-impermeable bactericidal antibiotic gentamicin to kill any extracellular bacteria. Cells were then washed three times with PBS, lysed in 1 mL of 0.1% Triton X-100, and plated on LB-agar plates. Bacteria present in these lysates, representing the number of bacteria present intracellularly, were tittered. Invasion frequencies were calculated as the number of bacteria surviving incubation with gentamicin divided by the total number of bacteria present just before addition of gentamicin [9].

### Detection of *E. coli *adhesin expression by reverse transcriptase-polymerase chain reaction (RT-PCR)

Total RNAs were isolated from J96 *E. coli *grown in pH 7.0 with no NaCl and in pH 5.5 with 400 mM NaCl LB medium by using TRIzol reagent as previous described [20]. The cDNAs used for PCR were each synthesized from total RNA by using the random hexamer primer from SSII RT kit (Invitrogen). 3 μg of cDNA was used as the template for amplification (30 cycles): denaturation at 94°C for 1 min, annealing at 65°C for 1 min, extension at 72°C for 2 min. The primers were used as follows: FimH forward primer, 5'-CAC TGC TCA CAG GCG TCA AA-3'; FimH reverse primer, 5'-GAT GGG CTG GTC GGT AAA TG-3'; papG forward primer, 5'-AAT ACA GGC TCT GCT ACA-3'; papG reverse primer, 5'-TTT CCC TCT TCA CCA TAC-3'; 16S rRNA forward primer, 5'-CTC CTA CGG GAG GCA GCA G-3'; 16S rRNA reverse primer, 5'-GWA TTA CCG CGG CKG CTG-3'.

### Detection of the invertible element orientation by limiting-dilution PCR analyses

Chromosomeal DNA from J96 *E. coli *grown in pH 7.0 with no NaCl and in PH 5.5 with 400 mM NaCl were isolated by using a GenomicPrep Cells DNA Isolation Kit according to the manufacture's instruction. The DNAs were standardized and used for PCR with the INV-FIMA primer pair to amplify the phase-on orientation of the invertible element and the FIMA-INV primer pair to amplify the phase-off orientation of the invertible element [21]. The chromosomal DNAs were each serially twofold diluted to a dilution of 1/32, and an aliquot of each dilution was then amplified. PCR was performed at least three times with three separate chromosomal DNA preparations for each type of growth conditions [3].

### Detection of IL-8 mRNA expression by real-time quantitative PCR

Total RNA preparation and the RT reaction were carried out as described previously [22]. PCRs were performed using an ABI Prism 7900HT according to the manufacturer's instructions. Amplification of specific PCR products was detected using the SYBR Green PCR Master Mix (Applied Biosystems). The designed primers in this study were: IL-8 forward primer, 5'-ACT GAG AGT GAT TGA GAG TGG AC-3'; IL-8 reverse primer, 5'-AAC CCT CTG CAC CCA GTT TTC-3'; 18S rRNA forward primer, 5'-CGG CGA CGA CCC ATT CGA AC-3'; 18S rRNA reverse primer, 5'-GAA TCG AAC CCT GAT TCC CCG TC-3'. RNA samples were normalized to the level of 18S rRNA. The real-time PCR was performed in triplicate in a total reaction volume of 25 μL containing 12.5 μL of SYBR Green PCR Master Mix, 300 nM forward and reverse primers, 11 μL of distilled H_2_O, and 1 μL of cDNA from each sample. Samples were heated for 10 min at 95°C and amplified for 40 cycles of 15 sec at 95°C and of 60 sec at 60°C. Quantification was performed using the 2^-ΔΔCt ^method [23], where Ct value was defined as threshold cycle of PCR at which amplified product was detected. The ΔCt was obtained by subtracting the housekeeping gene (18s rRNA) Ct value from the Ct value of the gene of interest (IL-8). The present study used ΔCt of control subjects as the calibrator. The fold change was calculated according to the formula 2^-ΔΔCt^, where ΔΔCt was the difference between ΔCt and the ΔCt calibrator value (which was assigned a value of 1 arbitrary unit).

### IL-8 enzyme-linked immunosorbent assay (ELISA)

The levels of IL-8 in the conditioned media were determined by using sandwich ELISA (sensitivity 18 pg/mL; R&D) according to manufacturer's protocols, as previously described [22].

### Western Blot Analysis

Cells were lysed with a buffer containing 1% NP-40, 0.5% sodium deoxycholate, 0.1% SDS, and a protease inhibitor mixture (PMSF, aprotinin, and sodium orthovanadate). The total cell lysate (50 μg of protein) was separated by SDS-polyacrylamide gel electrophoresis (PAGE) (12% running, 4% stacking) and analyzed by using the designated antibodies and the Western-Light chemiluminescent detection system (Bio-Rad, Hercules, CA), as previously described [24].

### siRNA transfection

For siRNA transfection, 5637 and 786-O cells were transfected with the designated siRNA by using RNAiMAX transfection kit (Invitrogen) [23].

### Transcription factor assays (TF ELISA assays)

Nuclear extracts of cells were prepared as previously described [24]. Equal amounts of nuclear extracts were used for quantitative measurements of Sp1 and AP-1 activation using commercially available ELISA kits (Panomics, Redwood City, CA) that measure p65 NF-κB and AP-1-DNA binding activities [23].

### Statistical Analysis

The results are expressed as mean ± standard error of the mean (SEM). Statistical analysis was determined by using an independent Student t-test for two groups of data and analysis of variance (ANOVA) followed by Scheffe's test for multiple comparisons. *P *values less than 0.05 were considered significant.

## Results

### Identification of fimbriae expressed in UPEC J96 under different conditions

**Figure 1:**
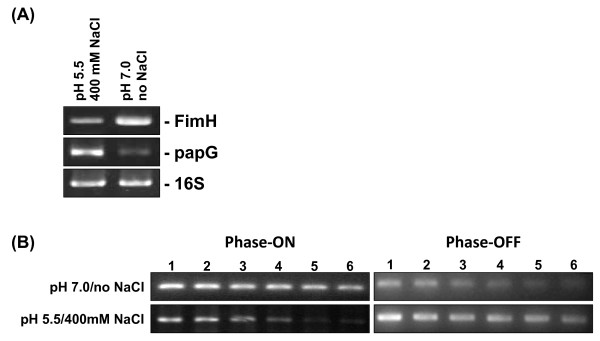
**Measurement of adhesin mRNA expression and invertible element orientation in UPEC J96 grown under different conditions**. (A) Analysis of adhesins FimH and PapG mRNA expression in J96 grown in either pH 7.0 with no NaCl medium or pH 5.5 with 400 mM NaCl medium. RNA samples from J96 were isolated and subjected to RT-PCR analysis. (B) The PCR was performed with chromosomal DNA isolated from J96 grown in pH 7.0 with no NaCl condition or in pH 5.5 with 400 mM NaCl condition, using the INV and FIMA primers to amplify phase-on-oriented DNA, and the FIME and INV primers to amplify phase-off-oriented DNA. The dilutions were used for PCR as follows: undiluted (lanes 1), 1/2 (lanes 2), 1/4 (lanes 3), 1/8 (lanes 4), 1/16 (lanes 5), 1/32 (lanes 6). All PCR products were separated by agarose gel electrophoresis and stained with ethidium bromide.

To address the question of whether pH and osmolarity affect the expression of type 1 and P type fimbriae, UPEC strain J96 was cultured in either a pH 7.0 with no NaCl added medium, or a pH 5.5 with 400 mM NaCl medium [3]. J96 cultured in pH 7.0 with no NaCl medium was positive on type 1 (as demonstrated by the mannose-sensitive hemagglutination (MSHA) of erythrocytes) but negative for P fimbriae (as demonstrated by a lack of agglutination of latex beads coated with the specific P fimbrial α-Gal(1-4)β-Gal receptor). In contrast, J96 cultured in pH 5.5 with 400 mM NaCl medium was negative for type 1 fimbriae, but positive on P fimbriae (Table 1). To confirm these results, the expression of fimbrial adhesin mRNA was determined. As shown in Figure 1A, J96 cultured in a pH 7.0 with no NaCl condition favored FimH mRNA expression, however, J96 cultured in a pH 5.5 with 400 mM NaCl condition favored papG mRNA expression.

**Table 1:** Type 1 and P fimbrial phenotypes of UPEC J96 grown in different environmental conditions.

	pH 7.0 with no NaCl		pH 5.5 with 400 mM NaCl	
	
	**MSHA**^*a*^	**Gal-Gal**^*b*^	MSHA	Gal-Gal
J96	+++	+	+	+++

HB101	--	--	--	--

^*a *^Absence (-) or relative present amount (+, +++) of MSHA indicates type 1 fimbrial phenotype.

^*b *^Absence (-) or relative present amount (+, +++) α-Gal(1-4)-β Gal-coated latex bead agglutination (Gal-Gal) indicates P fimbrial phenotype.

### Effects of pH and osmotic conditions on invertible element switching

To determine the orientation of the invertible element, PCR was performed by using chromosomal DNAs from J96 cultured in pH 7.0 with no NaCl medium, and pH 5.5 with 400 mM NaCl medium. The DNAs were serially twofold diluted and subjected to PCR analysis. Specific primer pairs for the phase-on and phase-off orientations were used [21]. There was a significant decrease in phase-on orientation when J96 grown in pH 5.5 with 400 mM NaCl condition (Figure 1B). Phase-off orientation also increased efficiently when pH 5.5 with 400 mM NaCl condition was compared to pH 7.0 with no NaCl condition (Figure 1B).

### Invasion of the uroepithelial cells by UPEC

**Figure 2:**
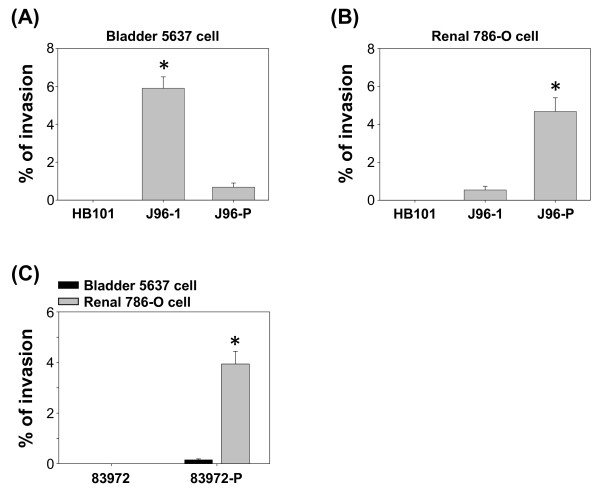
**Invasion of the uroepithelial cells by UPEC**. (A) Invasion percentage of bladder 5637 epithelial cells by J96 grown in either pH 7.0 with no NaCl medium (J96-1) or pH 5.5 with 400 mM NaCl medium (J96-P). * *p *< 0.05 vs. J96-P. (B) Invasion percentage of renal 786-O epithelial cells by either J96-1 or J96-P. * *p *< 0.05 vs. J96-1. (C) Invasion percentage of 5637 and 786-O epithelial cells by non-fimbriated *E. coli *83972 and P-fimbriated 83972 (83972-P). * *p *< 0.05 vs. 5637 cells infected by 83972-P.

The different roles of type 1 and P fimbriae in mediating bacterial invasion by uroepithelial cells were investigated using gentamicin protection assays [9]. UPEC strain J96 was cultured in either a pH 7.0 with no NaCl medium, or a pH 5.5 with 400 mM NaCl medium to induce specific fimbriae expression. Type 1 fimbriated J96 (J96-1) invaded bladder 5637 cells much more readily (Figure 2A), conversely, P type fimbriated J96 (J96-P) preferred renal 786-O epithelial cells (Figure 2B). The non-fimbriated HB101 failed to infect either epithelial cell.

To further confirm these results, the non-fimbriated *E. coli *strain 83972 and P-fimbriated 83972 (83972-P) were used. The functional fimbriae of *E. coli *83972 are not expressed in the urinary tract or after *in vitro *subculture [25]. 83972-P was derived by transformation with plasmid carrying genes encoding functional P fimbriae. The non-fimbriated 83972 failed to infect either epithelial cell, however, 83972-P infected renal 786-O cells effectively compared to bladder 5637 cells (Figure 2C). These data indicated that different types of fimbriae mediated UPEC invasion of their specific target cells.

### IL-8 gene expression and secretion after invasion of the uroepithelial cells by UPEC

**Figure 3:**
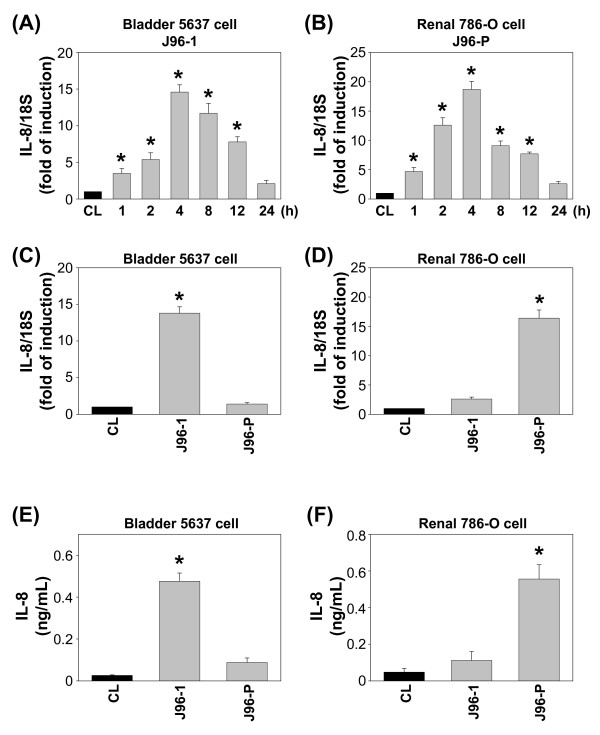
**Induction of IL-8 expression in uroepithelail cells by different fimbriated J96 invasion**. RNA samples were isolated at the indicated time periods with the infection with indicated fimbrial types, followed by subjecting to real-time PCR analysis. Data are normalized against 18S rRNA level and presented as fold changes in fluorescent density in comparison to that of control ECs (CL) (A-D). The IL-8 protein secretion in conditioned media was determined by ELISA analyses (E,F). 5637 or 786-O cells were kept as controls (CL) or invasion with either type 1 or P fimbriated J96 for the times indicated (A,B), or the cells were invaded with different fimbrial types of J96 for 4 h (C,E) or 12 h (D,F). Data are shown as mean ± standard error of the mean (SEM). * *P *< 0.05 versus control epithelial cells (CL).

We examined the effect of J96 invasion on the expression of IL-8 by human renal and bladder epithelial cells. Bladder 5637 cells were invaded by J96-1 (grown in pH7.0 with no NaCl medium), whereas renal 786-O cells were invaded by J96-P (grown in pH5.5 with 400 mM NaCl medium) for the times indicated. The changes in IL-8 mRNA expression were analyzed by real-time PCR normalized to house keeping gene 18S rRNA. The IL-8 mRNA levels in both 5637 and 786-O cells began to increase after 1 h of J96 invasion and reached its highest level at 4 h; thereafter it gradually reduced to a basal level after (Figure 3A for 5637 invaded by J96-1, Figure 3B for 786-O invaded by J96-P).

**Figure 4:**
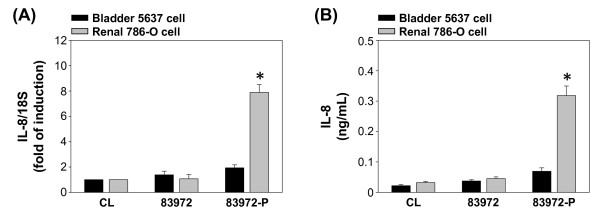
**Induction of IL-8 expression in uroepithelail cells by non-fimbriated E. coli 83972 and P-fimbriated 83972 (83972-P) invasion**. (A) 5637 or 786-O cells were kept as controls (CL) or invasion with non-fimbriated 83972 or 83972-P for 4 h, RNA samples were isolated and subjected to real-time PCR analysis. Data are normalized against 18S rRNA level and presented as fold changes in fluorescent density in comparison to that of control ECs (CL). (B) The IL-8 protein secretion in conditioned media was determined by ELISA analyses. 5637 or 786-O cells were kept as controls (CL) or invasion with non-fimbriated 83972 or 83972-P for 12 h. Data are shown as mean ± standard error of the mean (SEM). * *P *< 0.05 versus control epithelial cells (CL).

To determine whether IL-8 expression and secretion were dependent on specific J96/host cell interaction, both 5637 and 786-O cells were invaded by J96 with either type 1 or P type fimbriae. The results showed that invasion with J96-1 significantly increased IL-8 mRNA expression (Figure 3C) and protein secretion (Figure 3E), whereas J96-P had little effect on IL-8 expression/secretion in 5637 cells (Figures 3C and 3E). On the contrary, J96-P significantly increased IL-8 mRNA expression and protein secretion, whereas J96-1 had little effect on IL-8 expression/secretion in 786-O cells (Figures 3D and 3F). Similarly, 83972-P also increased IL-8 mRNA expression and protein secretion significantly in 786-O cells, but not 5637 cells (Figures 4A for mRNA expression and 4B for protein secretion). These results clear reveal the specific UPEC/host interaction is necessary for the regulation IL-8 gene expression in host cells.

### MAP kinase phosphorylation by J96 infection

**Figure 5:**
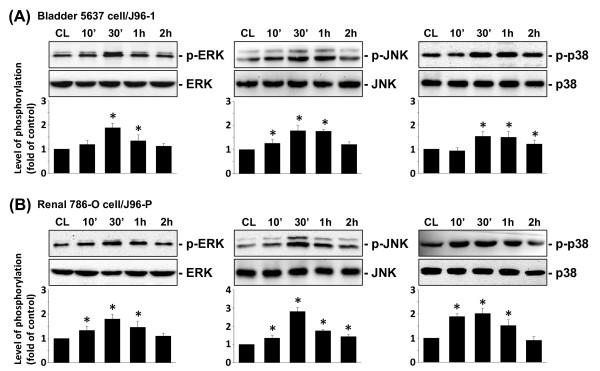
**Invasion with specific fimbriated J96 induces uroepithelial cells to increase the phosphorylation of ERK, JNK, and p38**. (A) 5637 cells were kept as controls (CL) or invaded with type 1 fimbriated J96 (J96-1), or (B) 786-O cells were kept as controls (CL) or invaded with P fimbriated J96 (J96-P) for the times indicated, and the phosphorylations of ERK, JNK, and p38 were determined by using Western blot analyses. Phosphorylated ERK, JNK, and p38 levels are presented as band densities (normalized to total protein levels) relative to CL. The results are mean ± SEM from at least 3 independent experiments. * *P *< 0.05 versus control EC (CL).

Members of the MAPK superfamily [i.e., ERK, JNK, and p38] are known to regulate gene expression and cellular functions [26]. The phosphorylation levels of ERK, JNK, and p38 in 5637 cells increased rapidly after invasion with J96-1, reaching maximal levels at 30 min (Figure 5A). In addition, phosphorylation levels of ERK, JNK, and p38 also increased in 786-O cells after invasion with J96-P, reaching maximal levels at 30 min (Figure 5B). After transient increases, the levels of MAPK phosphorylation in both 5637 and 786-O cells decreased to nearly basal levels.

### Effect of MAPK inhibitors on IL-8 expression in uroepithelail cells

**Figure 6:**
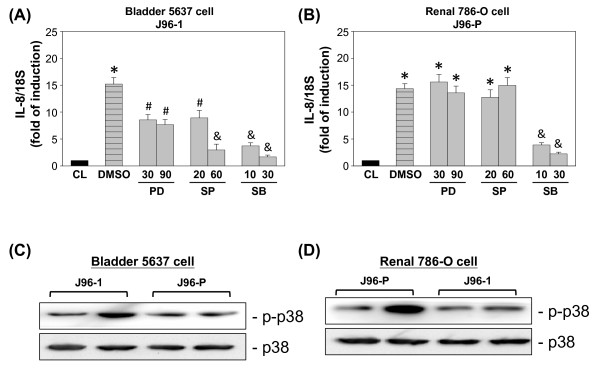
**Effect of MAPK inhibitors on the regulation of IL-8 expression in uroepithelail cells**. (A) 5637 cells were kept as controls (CL) or invaded with type 1 fimbriated J96 (J96-1) for 4 h. (B) 786-O cells were kept as controls (CL) or invaded with P fimbriated J96 (J96-P) for 4 h. Before being kept as controls or invaded with J96, cells were pretreated with PD98059 (PD), SP600125 (SP), or SB203580 (SB) separately for 1 h. Data are normalized against 18S rRNA level and presented as fold changes in comparison to control cells (CL) and. The results are shown as mean ± SEM. * *P *< 0.05 versus CL. ^#^*P *< 0.05 versus DMSO-treated cells with J96 invasion. ^&^*P *< 0.01 versus DMSO-treated cells with J96 invasion. (C) and (D) The phosphorylation of p38 in 5637 cells (C) and 786-O cells (D) after 30 min of either J96-1 or J96-P invasion was determined by using Western blot.

To determine whether the J96 invasion induced IL-8 expression is mediated through the MAPK-dependent pathway, both types of uroepithelial cells were incubated with the specific inhibitor for ERK (PD98059; 30 and 90 μM), JNK (SP600125; 20 and 60 μM), or p38 (SB203580; 10 and 30 μM) for 1 hour before and during infection with J96. The J96-1-induced IL-8 mRNA expression in 5637 cells were significantly inhibited by 60 μM SP600125 and SB203580, and partially inhibited by PD98059 or 20 μM SP600125 (Figure 6A). Treatment of 786-O cells with SB203590 results in significant inhibition of J96-P-induced IL-8 mRNA expression, but PD98059 or SP600125 had little effect (Figure 6B).

To investigate whether p38 phosphorylation was dependent on J96 invasion, both 5637 and 786-O cells were invaded by either J96-1 or J96-P. As shown in Figure 6C, J96-1 caused p38 phosphorylation after 30 min invasion, whereas J96-P had no effect on p38 phosphorylation in 5637 cells. In contrary to 5637 cells, only J96-P, but not J96-1, induced p38 phosphorylation in 786-O cells (Figure 6D). These results suggested that p38 phosphorylation in uroepithelial cells was dependent on specific J96 invasion.

### AP-1 mediated IL-8 expression by J96 invasion

**Figure 7:**
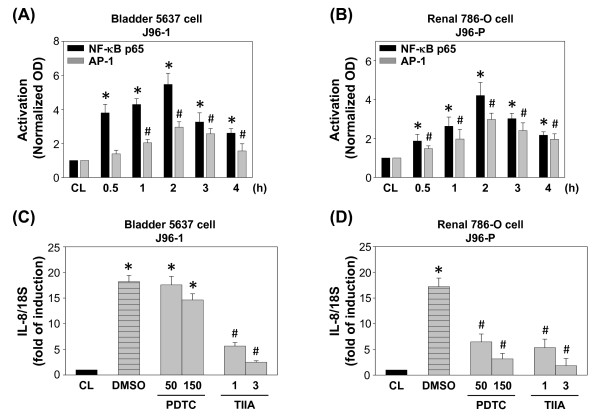
**J96 invasion induced p65 NF-κB and AP-1 binding activities**. (A) NF-κB and AP-1 activation by type 1 fimbriated J96 (J96-1) invasion in 5637 cells, and (B) NF-κB and AP-1 activation by P fimbriated J96 (J96-P) invasion in 786-O cells were determined by TF ELISA assay. All bar graphs represent folds of control cells (CL), mean ± SEM. * *P *< 0.05 versus p65 NF-κB activation in CL. ^#^*P *< 0.05 versus AP-1 activation in CL. (C) 5637 cells were kept as controls (CL) or invaded with J96-1 for 4 h. (D) 786-O cells were kept as controls (CL) or invaded with J96-P for 4 h. Before being kept as controls or invaded with J96, cells were pretreated with NF-κB inhibitor Pyrrolidine dithiocarbamate (PDTC), or AP-1 inhibitor Tanshinone IIA (TIIA) individually for 1 h. Data are normalized to 18S rRNA level and presented as fold changes in comparison to control cells (CL) and. The results are shown as mean ± SEM. * *P *< 0.05 versus CL. ^#^*P *< 0.05 versus DMSO-treated cells with J96 invasion. ^&^*P *< 0.01 versus DMSO-treated cells with J96 invasion.

To further investigate the regulation of IL-8 expression by J96, we studied the NF-κB and AP-1 transcription factor binding sites of the IL-8 promoter region [27]. Quantitative analyses for NF-κB p65 and AP-1 binding activities in vitro were performed by using TF ELISA kits from Panomics. We first showed that invasion of 5637 cells with J96-1 caused both p65 and AP-1-DNA binding activities to increase at 30 min and remain elevated for at least 2 h (Figure 7A). In addition, invasion of 786-O cells with J96-P also displayed similar results (Figure 7B). We then further tested whether NF-κB and AP-1 activations are involved in the signal transduction leading to the J96 invasion-induced IL-8 gene expression. 5637 cells were incubated with the specific inhibitors for NF-κB (PDTC, 50 and 150 μM) and AP-1 (Tanshinone IIA, 1 and 3 μM) for 1 h, and followed by infection with J96-1 for 4 h. The J96-1-induced IL-8 mRNA expression was significantly reduced by Tanshinone IIA inhibition (Figure 7C). However, the J96-P-induced IL-8 mRNA expression in 786-O cells was significantly attenuated by both PDTC and Tanshinone IIA (Figure 7D). These data indicated that AP-1 was mainly involved in the regulation of J96-induced IL-8 gene expression in both bladder and renal epithelial cells, whereas NF-κB was also involved in the regulation of J96-1-induced IL-8 expression in renal epithelial cells.

### p38 MAPK is involved in J96-induced AP-1 activation

**Figure 8:**
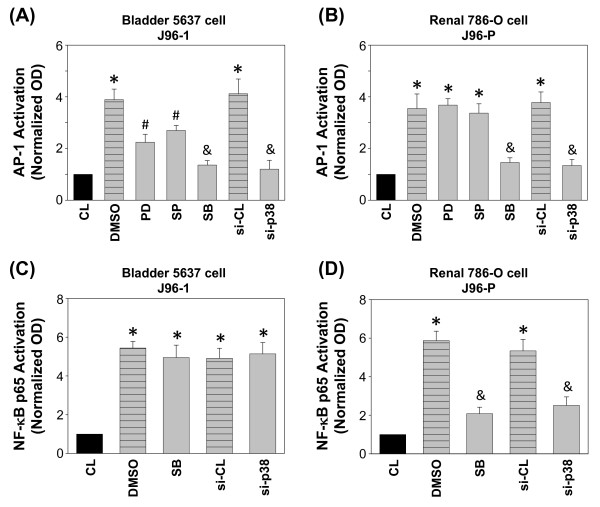
**MAPK signaling pathways were involved in J96-induced AP-1 activation of uroepithelial cells**. Type 1 fimbriated J96 (J96-1) induced AP-1 (A) and NF-κB (C) activation in 5637 cells, and P fimbriated J96 (J96-P) induced AP-1 (B) and NF-κB (D) activation in 786-O cells were determined by TF ELISA assays in cells pretreated with vesicle (DMSO), PD98059 (PD; 30 μM), SP600125 (SP; 20 μM), or SB203580 (SB; 10 μM) individually for 1 h, or transfected with si-CL, si-p38, and then invaded with J96 for 2 h. The results are shown as mean ± SEM. * *P *< 0.05 versus CL. ^#^*P *< 0.05 versus DMSO-treated cells with J96 invasion. ^&^*P *< 0.01 versus DMSO-treated or si-CL transfected cells with J96 invasion.

To elucidate the roles of MAPKs in regulating AP-1 transcriptional activation, both 5637 and 786-O cells were pretreatment with MAPK inhibitors or transfection with p38 siRNA followed by J96 invasion, and the AP-1 activation were assessed by AP-1 TF ELISA kits. 5637 cells pretreatment with PD98059 and SP600125 partially inhibited J96-1-induced AP-1-DNA binding activity, whereas pretreated with SB203580, or transfection with p38 siRNA significantly inhibited the J96-1-induced AP-1-DNA binding activity (Figure 8A). 786-O cells pretreatment with SB203580 or transfection with p38 siRNA also inhibited J96-P-induced AP-1-DNA binding activity (Figure 8B). J96-P-induced NF-κB-DNA binding activity was also affected by SB203580 or p38 siRNA in 786-O cells (Figure 8D); however, SB203580 and p38 siRNA had no effect on J96-1-induced NF-κB-DNA binding activity in 5637 cells (Figure 8C).

## Discussion

**Figure 9:**
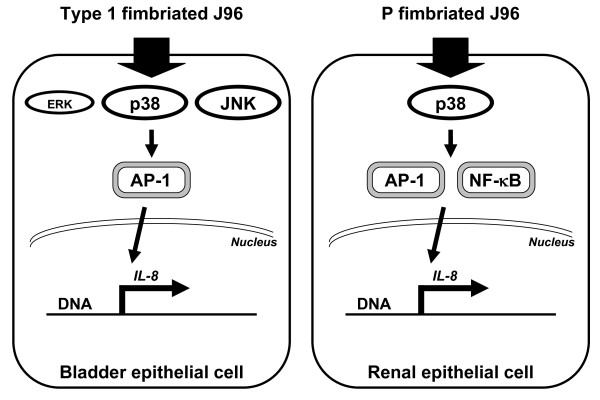
**Schematic representation of the signaling pathways regulating the expressions of IL-8 in uroepithelial cells in response to different fimbrial types of J96 invasion**.

This study examined multiple aspects of the environment/bacteria/host cells interactions: environmental conditions regulation of bacterial phenotypes, which leads to the specific host cell interactions, the differential signaling events, and consequential gene expression. We found that an environment with a high pH combined with low osmolarity (pH 7.0 with no NaCl) favorable for type 1 fimbrial expression, whereas low pH combined with high osmolarity (pH 5.5 with 400 mM NaCl) favorable for P type fimbrial expression in UPEC J96. Type 1 fimbriated J96-induced IL-8 expression was via ERK, JNK, p38 MAPK phosphorylation and AP-1 activation in bladder epithelial cells. Conversely, P fimbriated J96-induced IL-8 expression was through p38 MAPK phosphorylation and both NF-κB and AP-1 activation in renal epithelial cells (Figure 9).

Expression of the individual fimbriae in UPEC is regulated in response to growth conditions, and most are subject to phase variation [11,28]. The expression of type 1 fimbriae is controlled by a promoter situated on an invertible element of *fim *gene cluster, also referred to as the *fim *switch [11]. Type 1 fimbriae are expressed when the promoter faces phase-on direction. When the promoter faces the opposite orientation, *fim *gene cluster of UPEC are phase off and no type 1 fimbrial expression. The inversion of the *fim *switch is mediated by the recombinases FimB and FimE. FimB promotes inversion in both on-to-off and off-to-on directions, whereas FimE mediates predominantly on-to-off inversion [29]. Previous work has demonstrated that expression of PapB, a regulator from the P-fimbrial gene cluster, is able to prevent inversion of the *fim *switch [30]. Cross-talk with PapB has been shown to inhibit FimB-promoted recombination together with increasing *fimE *expression [11]. These results imply that PapB produced from an activated P-fimbrial gene cluster can act on the *fim *locus to prevent expression of type 1 fimbriae. In addition, different fimbrial expression patterns are known to depend on environmental and growth conditions. Schwan et al. have demonstrated that combination of pH and osmolarity have a major impact on *fim *gene expression in UPEC strains [4]. Our results also show that expression of different fimbriae is dependent on the environmental stimuli (i.e. pH and osmolarity) received by the UPEC strain J96. This result indicates that the environmental controls designate J96 to expresses mainly one fimbrial type at a time.

The ability of UPEC to infect urinary tract depends on its ability to express fimbriae that facilitate colonization on the uroepithelial cell surfaces. The cross-talk between different fimbrial gene systems is presumably important for pathogens to survive under changing environmental conditions. P fimbriae are produced by pyelonephritic strains of UPEC and mediate binding to glycolipids that predominate in the kidney. Consequently, P fimbriae have been shown to be critical for UPEC to cause pyelonephritis [31]. Type 1 fimbriae bind to glycoproteins present on the bladder epithelial surface and are critical for the establishment of cystitis [32]. UPEC strains expressing either P or type 1 fimbriae have been demonstrated to augment bladder and renal epithelial cell cytokine production compared to isogenic afimbriated strains [33,34]. In the present study, our data have demonstrated that the ability of type 1 fimbriated J96 to invade bladder epithelial cells and P fimbriated J96 or 83972 to invade renal epithelial cells are the key events for the induction of IL-8 expression. In addition, it has been shown that bacterial attachment mediated by different adhesive fimbriae results in the activation of distinct signaling pathways [35]. The binding of P fimbriated UPEC to renal epithelial cells appears to activate IL-6 expression via a Toll-like receptor 4 (LTR4)-dependent, lipopolysaccharide (LPS)-independent mechanism [36]. However, studies with bladder epithelial cells demonstrated that LPS is the primary bacterial factor activating cytokine production, and that type 1 fimbriae augment the presentation of LPS to LPS receptor on the bladder epithelial cells [34]. Although the importance of fimbriated UPEC in the pathogenesis of UTI has been suggested, the exact mechanism has not been fully understood. Many inflammatory genes are induced by NF-κB, AP-1, and MAPK activation has been shown to be important for the NF-κB/AP-1-mediated inflammatory process. The MAPK family kinases (e.g., p38, JNK, and ERK) are involved in the induction of IL-8 transcription, although the contribution of each kinase varies depending on cell type and stimulus [16,19]. The present study demonstrated that in bladder epithelial cells, AP-1, but not NF-κB, activation upon type 1 fimbriated J96 invasion was required for IL-8 expression. Such an AP-1 activation required JNK and p38 phosphorylation (whereas ERK is also involved, but play lesser roles) in type 1 fimbriated J96-induced AP-1 activation and IL-8 expression. Conversely, in renal epithelial cells, the results showed that p38 phosphorylation (whereas ERK and JNK are not involved) was also required for P fimbriated J96 activation of both NF-κB and AP-1 and the consequential IL-8 expression.

In conclusion, we have shown that the specific interactions between different fimbriated UPEC (J96) with distinct human uroepithelial cells to induce IL-8 expression via diverse signaling pathways. UPEC-mediated induction of proinflammatory cytokines (such as IL-8) in human uroepithelial cells provides evidence of UPEC inflammatory activities, which may lead to its pathogenic reactions.

## Competing interests

The authors declare that they have no competing interests.

## Authors' contributions

KWT and CNC designed research; KWT, HTL, TCT, YCW, YTY, KYC and CMC performed research; KWT and CNC analyzed data; and YSJL and CNC wrote the paper.
